# The independent effects of sleep fragmentation and physical activity on global cognition in healthy older adults

**DOI:** 10.1002/alz70860_099461

**Published:** 2025-12-23

**Authors:** Tara Kuhn, Laura E. Middleton, Lei Yu, David A. A. Bennett, Aron S Buchman, Andrew Lim

**Affiliations:** ^1^ University of Waterloo, Waterloo, ON, Canada; ^2^ Rush University Medical Center, Chicago, IL, USA; ^3^ University of Toronto, Toronto, ON, Canada

## Abstract

**Background:**

Better sleep and higher physical activity have been linked to better cognitive function with aging. Emerging evidence shows that physical activity and sleep may interact together to support aspects of cognition such as memory and executive functioning. However, it is less certain if physical activity and sleep work together to support cognition globally.

**Method:**

From the Rush Memory and Aging Project, 1171 older adults without known dementia were included in this analysis. Physical activity and sleep fragmentation were measured using actigraphy. Global cognitive function was assessed using a composite score created from a 19‐test cognitive battery. Multiple linear regression models were used to look at the cross‐sectional association between sleep, physical activity, and global cognition. Several models were used to look at the 1) individual effects of sleep and physical activity in separate models, 2) the independent effects of sleep and physical activity in the same model, and 3) the interactive effects of sleep and physical activity. All base models controled for age at visit, sex, and education, and additional models controlled for vascular health and vascular risk factors.

**Result:**

When examined individually, physical activity was associated with better global cognition (*b* = 0.04, 95% CI = 0.01 – 0.07, *p* < .01). In contrast, increased sleep fragmentation was associated with worse global cognition. (*b* = ‐0.02, 95% CI = ‐0.05 – ‐0.001, *p* < .05). When both sleep fragmentation and physical activity were considered in the same model, these relationships remained similar. Finally, when adding an interaction term between physical activity and sleep, the interaction was non‐significant (*b* = ‐0.01, 95% CI = ‐0.03 – 0.02, *p* = 0.53). All models remained significant when controlling for vascular health and vascular risk factors.

**Conclusion:**

Sleep and physical activity may have independent, but not interactive, impacts of global cognitive functioning. Physical activity may support better cognitive health, while having fragmented sleep may negatively impacts it. Further research is required to examine the longitudinal relationship between sleep, physical activity, and cognitive health.

